# SPACIA1/SAAL1 Deletion Results in a Moderate Delay in Collagen-Induced Arthritis Activity, along with mRNA Decay of *Cyclin-dependent Kinase 6* Gene

**DOI:** 10.3390/ijms19123828

**Published:** 2018-11-30

**Authors:** Ryoji Fujii, Rie Komatsu, Tomoo Sato, Iwao Seki, Koji Konomi, Hiroyuki Aono, Hisateru Niki, Kazuo Yudoh, Kusuki Nishioka, Toshihiro Nakajima

**Affiliations:** 1 Institute of Medical Science, St. Marianna University School of Medicine, Kanagawa 216-8512, Japan; komatsur@marianna-u.ac.jp (R.K.); tomoo@marianna-u.ac.jp (T.S.); yudo@marianna-u.ac.jp (K.Y.); marlin@tokyo-med.ac.jp (T.N.); 2 AYUMI Pharmaceutical Corporation, Kyoto 612-8374, Japan; iwao.seki@ayumi-pharma.com (I.S.); hiroyuki.aono@ayumi-pharma.com (H.A.); 3 Santen Pharmaceutical Co., Ltd., Osaka 533-8651, Japan; koji.konomi@santen.com; 4 Department of Orthopedic Surgery, St. Marianna University School of Medicine, Kanagawa 216-8511, Japan; h2niki@marianna-u.ac.jp; 5 Global Health Innovation Policy Program (GHIPP), National Graduate Institute for Policy Studies (GRIPS), Tokyo 106-8677, Japan; kusuki-nishioka@bz04.plala.or.jp; 6 Institute of Medical Science, Tokyo Medical University, Tokyo 160-8402, Japan; 7 Misato Marine Hospital, Kochi 781-0112, Japan

**Keywords:** cell cycle, collagen-induced arthritis, cyclin-dependent kinase, proliferation, rheumatoid arthritis, synovitis

## Abstract

This study was performed to elucidate the molecular function of the synoviocyte proliferation-associated in collagen-induced arthritis (CIA) 1/serum amyloid A-like 1 (SPACIA1/SAAL1) in mice CIA, an animal model of rheumatoid arthritis (RA), and human RA-synovial fibroblasts (RASFs). *SPACIA1/SAAL1*-deficient mice were generated and used to create mouse models of CIA in mild or severe disease conditions. Cell cycle-related genes, whose expression levels were affected by *SPACIA1/SAAL1* small interfering RNA (siRNA), were screened. Transcriptional and post-transcriptional effects of *SPACIA1/SAAL1* siRNA on cyclin-dependent kinase (cdk) *6* gene expression were investigated in human RASFs. *SPACIA1/SAAL1*-deficient mice showed later onset and slower progression of CIA than wild-type mice in severe disease conditions, but not in mild conditions. Expression levels of *cdk6*, but not *cdk4*, which are D-type cyclin partners, were downregulated by *SPACIA1/SAAL1* siRNA at the post-transcriptional level. The exacerbation of CIA depends on SPACIA1/SAAL1 expression, although CIA also progresses slowly in the absence of SPACIA1/SAAL1. The CDK6, expression of which is up-regulated by the SPACIA1/SAAL1 expression, might be a critical factor in the exacerbation of CIA.

## 1. Introduction

Rheumatoid arthritis (RA) is a chronic autoimmune disease characterized by synovial inflammation and hyperplasia [1,2]. Activated synovial fibroblasts, a major component of the hyperplastic synovial lining, are important in the pathogenesis of synovitis because they secrete cytokines and chemokines, leading to exacerbation of inflammation. Immuno-suppressive biologics such as tumor necrosis factor (TNF) inhibitors with methotrexate (MTX) have become an important part of the standard care for RA [3]. Folate antagonism by MTX is well known to exert anti-proliferative effects, but the administration of folic or folinic acid with MTX does not diminish the anti-inflammatory potential. Therefore, it is assumed that the anti-proliferative effects of MTX may not work in RA [4]. On the contrary, other studies have shown the potential of an anti-proliferative strategy using a kinase inhibitor of both cyclin-dependent kinase (CDK) 4 and CDK6, cyclin D partners in the cell cycle, in some mouse models of RA [5,6]. We believe that the abnormal synovial proliferation in RA should be a crucial target for drug discovery.

In our previous study, we identified synoviocyte proliferation-associated in collagen-induced arthritis (CIA) 1/serum amyloid A-like 1 (*SPACIA1/SAAL1*) as an overexpressed gene in the joint tissue of mice with CIA and indicated that the gene was associated with abnormal synovial proliferation in human RA [7]. We also showed that *SPACIA1/SAAL1* small interfering RNA (siRNA) inhibited the proliferation of RA synovial fibroblasts (RASFs) in vitro and that *SPACIA1/SAAL1* transgenic mice exhibited early onset and rapid progression of CIA. These findings suggested that SPACIA1/SAAL1 could be involved in the progression of synovitis. In the present study, we generated *SPACIA1/SAAL1*-deficient mice and observed the molecular function of SPACIA1/SAAL1 in mice CIA and human RASFs.

## 2. Results

### 2.1. Generation of SPACIA1-Deficient Mice

**Figure 1:**
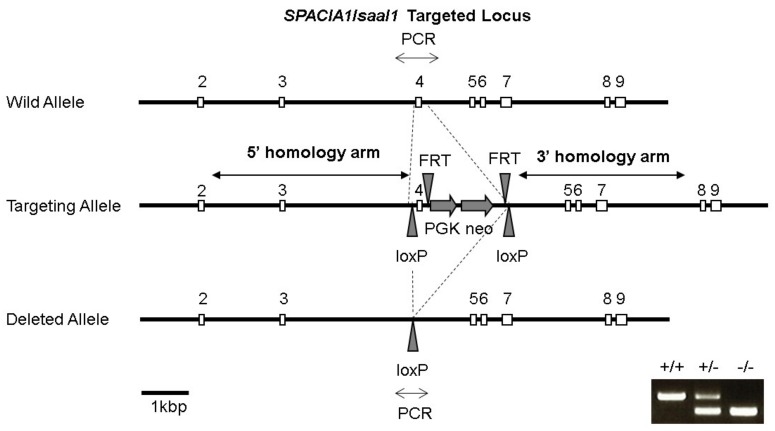
Schematic depiction of the *SPACIA1/SAAL1* gene-targeting strategy. Homologous recombination resulted in exon 4 being flanked by loxP sites; deletion was achieved by Cre recombinase-mediated excision. The “^+/+^”, “^+/−^” and “^−/−^” indicate wild-type (wt), heterozygous and homozygous mice for the *SPACIA1/SAAL1* gene.

A schematic representation of the *SPACIA1/SAAL1* targeted locus to generate *SPACIA1*-deficient mice is shown in Figure 1. The deletion of exon 4 in the *SPACIA1*-deficient mice was confirmed by polymerase chain reaction (PCR) analysis (Figure 1). Heterozygous mice (*SPACIA1*^+/−^) and even homozygous (*SPACIA1*^−/−^) lacking SPACIA1 systemically developed normally and did not show obvious abnormalities.

### 2.2. Progression of CIA in SPACIA1-Deficient Mice

**Figure 2:**
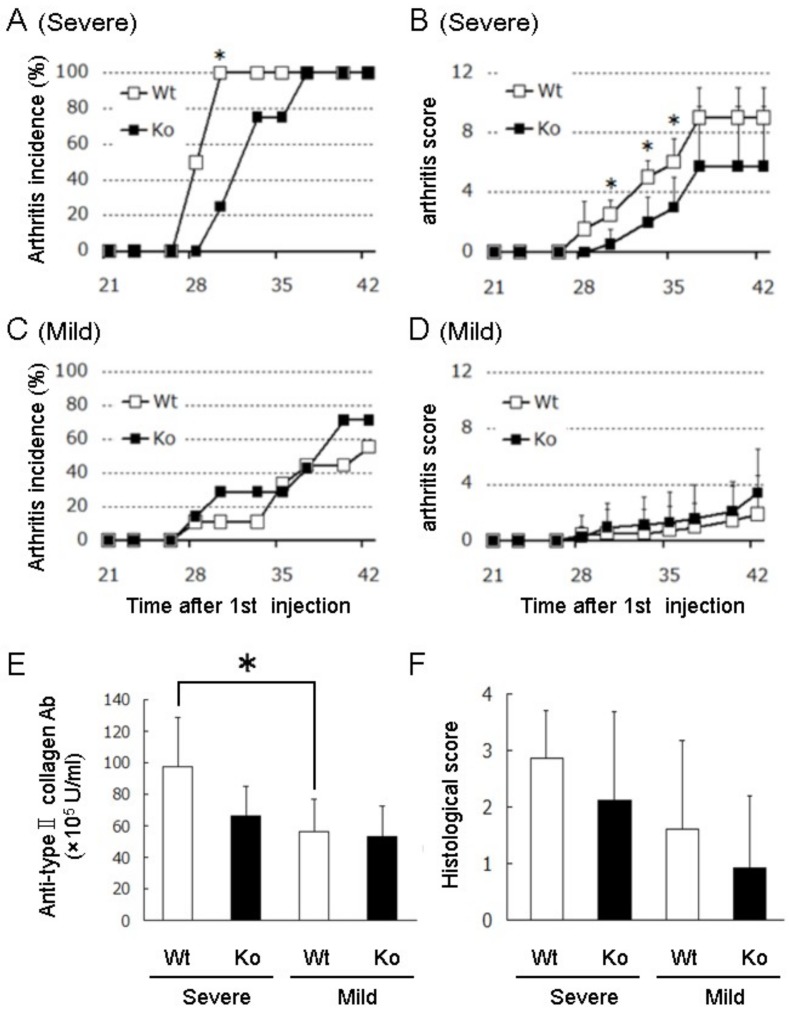
Incidence of arthritis (**A,C**) and mean arthritis score (**B,D**) in *SPACIA1*-deficient (ko) or wt mice injected with “Severe” or “Mild” collagen II (CII) are shown (wt; *n* = 4, ko; *n* = 5 (**A,B**), wt; *n* = 9, ko; *n* = 7 (**C,D**)). Data points and error bars correspond to the mean and SD, respectively. * *p* < 0.05. (**E**,**F**) Anti-CII antibody levels (**E**) and histopathological scores of the knee joints (**F**) in *SPACIA1*-deficient or wt mice treated with the “Severe” or “Mild” effective lot of CII. The presented data are from a representative experiment that was repeated three times with similar results. * *p* < 0.05.

In the previous study using SPACIA1 overexpressing mice, we reported that SPACIA1 is a synovitis modifying factor [7]. In this study, to confirm whether SPACIA1 is essential in the progression of synovitis, arthritis was induced using collagen in the *SPACIA1*-deficient mice (Figure 2). In CIA, different effects on the incidence and arthritis score are usually observed among the different production lots of bovine type II collagen (CII). We administered highly- or moderately-effective lots of CII to *SPACIA1*-deficient and wild-type (wt) mice. The highly effective lot of CII (labeled “Severe” in Figure 2) eventually caused an arthritis incidence of 100% (Figure 2A) and a high arthritis severity in the wt mice (Figure 2B). On day 30 after the 1st injection, the arthritis incidence of the *SPACIA1*-deficient mice was significantly reduced compared with that of the wt mice (Figure 2A). On days 30 to 35 after the 1st injection, the arthritis severity of the *SPACIA1*-deficient mice was also significantly reduced (Figure 2B). However, no significant differences in arthritis incidence, arthritis severity, antibody titers against bovine CII or histological score were observed on day 42 (Figure 2A,B,E,F). On the other hand, the moderately-effective lot of CII (labeled “Mild” in Figure 2) ultimately resulted in an arthritis incidence of 71% and 56% in *SPACIA1*-deficient and wt mice, respectively (Figure 2C). The arthritis severity was also moderate (Figure 2D). Under these conditions, no significant differences were observed in arthritis incidence, arthritis severity, antibody titers against bovine CII or histological score between *SPACIA1*-deficient and wt mice (Figure 2C–F). SPACIA1 was therefore not essential in the progression of synovitis.

### 2.3. Identification of Cell Cycle Regulators Altered at the mRNA Expression Level by SPACIA1

**Figure 3:**
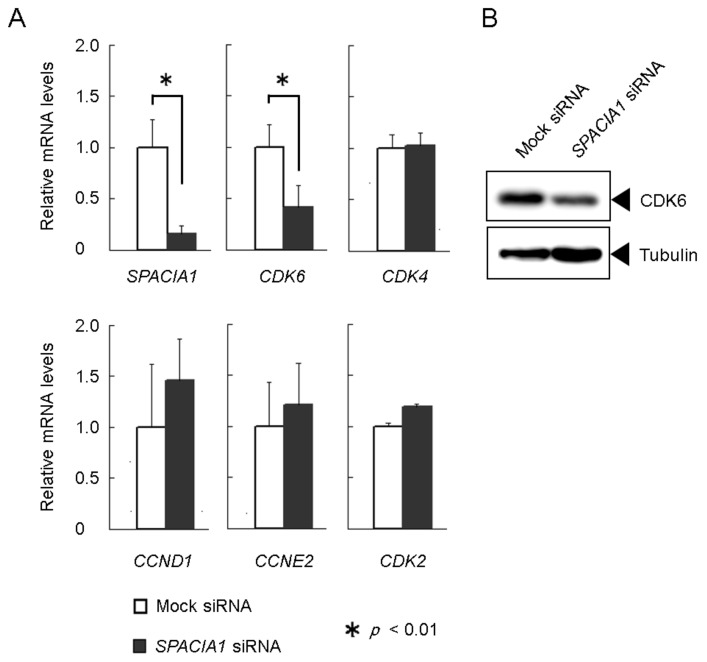
(**A**) The mRNA levels of G1 phase-related genes in human rheumatoid arthritis (RA)-synovial fibroblasts (RASFs) with *SPACIA1*-siRNA or mock siRNA. All experiments were performed in triplicate. (**B**) The protein expression levels of CDK6 in RASFs with *SPACIA1*-siRNA or mock siRNA. The alpha-tubulin expression levels are shown as the internal control. (**A,B**) The presented data are from a representative experiment that was repeated three times with similar results.

In our previous study, we reported that SPACIA1 is highly expressed in the hyperplastic synovial lining from patients with RA, and could relate RA synovial fibroblast proliferation at the first growth (G1) phase of cell cycle [7]. To identify the cell cycle regulators that are affected by the SPACIA1 expression, we measured the mRNA expression of G1 phase-related genes in RASFs transfected with *SPACIA1* siRNA, compared with mock siRNA. *SPACIA1* siRNA effectively reduced *SPACIA1* mRNA expression in RASFs (Figure 3A). Only the expression of *cdk6* gene was altered two-fold or more by *SPACIA1* siRNA in the G1 phase-related genes that we picked up. The log2 ratio of *cdk6* was −1.6 (Table 1). These results were confirmed by quantitative real time-PCR (qRT-PCR). The mRNA expression of *cdk6* was reduced to half by SPACIA siRNA in RASFs, while that of CDK4, CCN (cyclin) D1, CCNE2 and CDK2 were not significantly altered (Figure 3A). *SPACIA1* siRNA also reduced the expression of CDK6 at the protein level (Figure 3B).

### 2.4. Expression of CDK6 Protein in Human RA-Synovial Tissue

**Figure 4:**
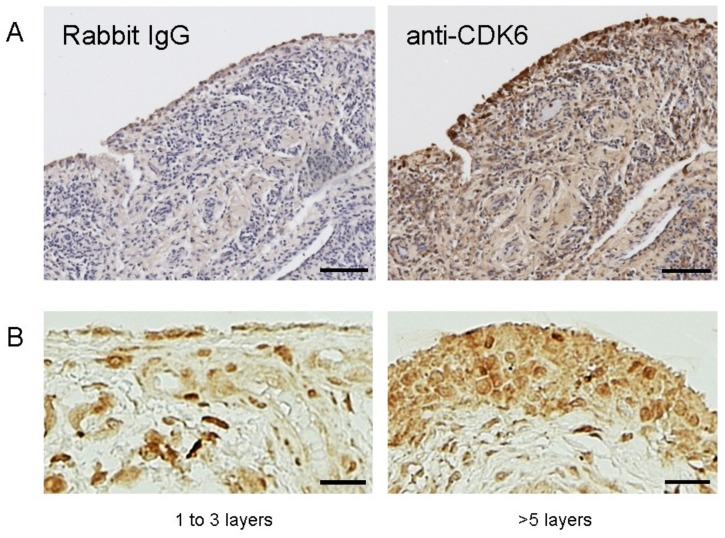
(**A**) Expression of CDK6 in human synovium. CDK6-positive cells (brown) are stained with 3,3′-diaminobenzidine. Mayer’s hematoxylin was used as a counterstain. Normal rabbit immunoglobulin G (IgG) was used as the negative control. Scale bar = 100 μm (**B**) Expression of CDK6 in human synovium with (>5 layers) or without (1 to 3 layers) hyperplasia of the synovial lining. Scale bar = 20 μm. Original magnification is 50× (**A**) and 200× (**B**). The presented images are from a RA patient. Similar results were obtained from two different RA patients.

To confirm the expression of the CDK6 protein in human RA-synovitis tissues in vivo, we performed immunohistochemistry, using synovitis tissues from RA patients. Anti-CDK6 antibody-specific signals were observed in RA-synovitis tissues, compared with the control of rabbit immunoglobulin G (IgG) (Figure 4A). More intense staining with the anti-CDK6 antibody was observed in multiple (>5) layers of synovial tissue (Figure 4B).

### 2.5. SPACIA1 Expression Affected cdk6-mRNA Stability, but Not Proximal Promoter Activity of cdk6 Gene

**Figure 5:**
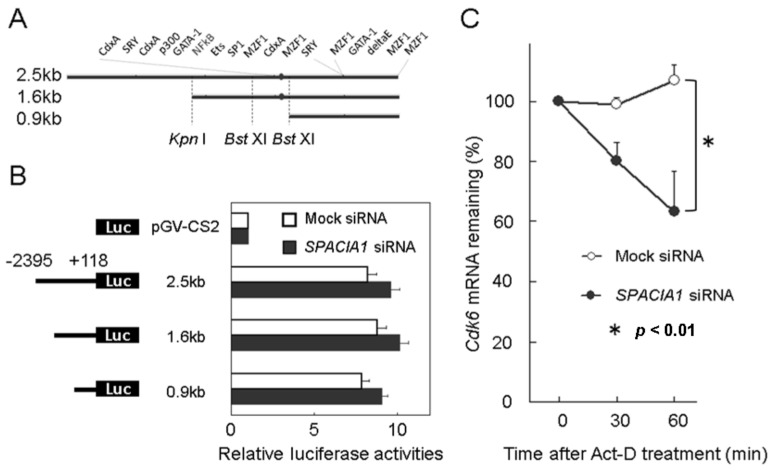
(**A**) Schematic representation of the *cdk6* proximal promoter region with restriction enzyme and putative transcription factor binding sites. The dots indicate the putative NF-kappa B binding site. (**B**) The proximal promoter activities of each construction in RASFs with *SAPCIA1*- or mock siRNA. RASFs were transfected with *SPACIA1*- or mock siRNA for 72 h. Subsequently, 24 h after transfection of each reporter vector, the promoter activity was measured using the Luciferase Assay System. (**C**) Messenger RNA stability analysis of the cdk6 gene. RASFs were transfected with *SPACIA1*- or mock siRNA for 72 h. The cells were harvested 0, 0.5, and 1 h after the addition of Actinomycin D (Act-D). The mRNA levels were measured by quantitative real time-polymerase chain reaction (qRT-PCR). (**B,C**) All experiments were performed in triplicate. The presented data are from a representative experiment that was repeated three times with similar results.

To identify the step in *cdk6* gene-expression that is affected by the SPACIA1 expression, we first performed reporter assays, using the proximal promoter of the *cdk6* gene in RASFs. The *cdk6*-promoters (Figure 5A) that we tested (2.5, 1.6 and 0.9 kb DNA fragments upstream of the *cdk6*-start codon) showed almost equal promoter activities in RASFs, and these activities showed an approximately 8-fold increase compared with the control vector (pGV-CS2). However, no alterations caused by *SPACIA1*-siRNA were observed, at least in these promoters (Figure 5B). Second, we performed mRNA stability assays. *SPACIA1* siRNA significantly reduced *cdk6*-mRNA stability compared with mock siRNA. Sixty minutes after Actinomycin-D treatment, the amount of *cdk6*-mRNA with *SPACIA1* siRNA was reduced to approximately 60%, compared with mock siRNA (Figure 5C). SPACIA1 related to *cdk6*-mRNA expression at the post-transcriptional level.

## 3. Discussion

In our previous study, we employed the mild (modified) form of CIA, using half the amount of CII for the 2nd injection, in transgenic mice overexpressing SPACIA1, because no significant alterations were observed in the severe (general) form of CIA, compared with wt mice [7]. In mild CIA, SPACIA1-overexpressing mice showed early onset and rapid progression of CIA. These results indicate that endogenous SPACIA1 expression in wt mice reached a plateau in severe CIA, but not in mild CIA. We, therefore, concluded that overexpression of SPACIA1 accelerates the progression of synovitis. In this study, we also employed both severe and mild forms of CIA in *SPACIA*-deficient mice. In severe CIA, *SPACIA1*-deficient mice showed later onset and slower progression of CIA than wt mice, indicating that the rapid progression of CIA in wt mice depends on SPACIA1 expression. Meanwhile, in the mild CIA, *SPACIA1*-deficient mice did not show any significant alterations compared to wt mice (Figure 2C–F). This suggests that CIA might progress independently of the SPACIA1 expression, even in wt mice. Summarizing the above, we reconfirmed that SPACIA1 is an exacerbation factor of CIA, although CIA also progresses slowly in the absence of SPACIA1. Some exacerbation factors that are induced by the SPACIA1 expression, such as CDK6, should be also induced by the other mechanism without SPACIA1. For instance, TNFα signaling induces cdk6 gene expression in Michigan Cancer Foundation 7 (MCF7) cells, a human breast cancer cell line [8]. Therefore, we have explored such factors that are induced by SPACIA1, not SPACIA1 itself, as drug targets in RA.

As we previously reported [7], SPACIA1 was screened as a factor suspected of showing altered expression in mouse CIA and affecting proliferation of cultured RASFs and was confirmed to be implicated in the G1 phase progression of RASFs. In this study, we screened for G1 phase-related genes, the expression of which was affected by the SPACIA1 expression and identified the *cdk6* gene (Table 1 and Figure 3). SPACIA1 was implicated in the decay of *cdk6* mRNA (Figure 5C) but did not affect proximal promoter activity (Figure 5B). SPACIA1 might thus be a factor regulating the mRNA stability of some inflammation-related genes. In future work, we intend to identify the SPACIA1-responsible element on *cdk6* mRNA.

Synovial proliferation is important in the pathology of RA, as well as abnormal immune responses and inflammation, and therefore constitutes a suitable drug target. However, almost no anti-proliferative agents have been developed to treat RA, while there are many immunosuppressive and anti-inflammatory therapies. Folate antagonism is well known as the antiproliferative effect of MTX. However, other groups have shown that administration of folic or folinic acid with MTX does not diminish the anti-inflammatory potential. This result suggests that the anti-proliferative effect of MTX might not be a major mechanism in RA treatment [4]. Furthermore, Thomas S. et al. reported MTX is a JAK/STAT inhibitor [9], not an inhibitor of AP1 and NF-κB pathways that activate proliferation. Kohsaka et al. also focused on anti-proliferative agents in RA treatment [5,6]. Their anti-CDK 4/6 agent is effective and has a synergistic effect with anti-TNF agents in some animal models of RA. Incidentally, double deficiency of both *cdk4* and *cdk6* results in either late embryonic or postnatal lethality, while single deficiency of *cdk4* or *cdk6* does not [10]. In fact, administration of an overdose of anti-CDK 4/6 agent causes cytopenia as previously described [6]. In this study, *SPACIA1* siRNA down-regulated CDK6, but not CDK4, in RASFs. Single inhibition of CDK6, but not CDK4, may have the potential for the treatment of RA, without causing side effects such as cytopenia. In future work, we aim to validate the potential of anti-CDK6 agents in mouse RA models.

## 4. Materials and Methods

### 4.1. Animals

Mice were raised under conventional conditions at our own facilities. Tap water and food were provided ad libitum.

### 4.2. Gene Targeting in ES Cells and Generation of the SPACIA1-Deficient Mouse Line

Generation of the *SPACIA1*-deficient mouse line was carried out by Ozgene Pty Ltd. (Bentley, Australia). Briefly, to ablate the functional mouse *SPACIA1/SAAL1* locus, we targeted its exon 4 for deletion using both the flippase/flippase recogition target (FLP/frt) and causes recombinase/locus of crossover (x) in P1 (CRE/loxP) systems to cause a frame-shift mutation (Figure 1). The mouse *SPACIA1/SAAL1* locus was generated by PCR from C57BL/6 mouse genomic DNA and its sequence was confirmed. The targeting construct was linearized and transfected into embryonic stem (ES) cells by electroporation. Recombinant ES cell clones were selected using G418 and injected into C57BL/6 mouse-derived blastocysts. The exon 4 and neomycin selection cassette was flanked by loxP recombination sites and excised in vivo by crossing with the Oz-Cre deleter strain (Ozgene). Finally, the *SPACIA1*-deficient mice were backcrossed onto the DBA/1J, a DBA/1 mouse strain distributed from The Jackson Laboratory, for 10 or more generations.

### 4.3. Induction and Assessment of CIA

To induce CIA in *SPACIA1*-deficient mice, two different production lots of CII were used. One lot of the collagen caused severe arthritis (Figure 2A,B), while the other caused mild arthritis (Figure 2C,D). Mice were injected into the base of the tail with 100 µg CII (highly- or moderately-effective lot) per mouse for both 1st and 2nd injection. Then, the arthritis score was assessed three times per week using Hughes’s scoring system [11]. All of the mice were sacrificed by decapitation, and then samples of their blood were collected to measure the antibody titers against bovine CII and the knee joints were harvested for histological analysis.

### 4.4. Gene Expression Analysis

To analyze the effect of *SPACIA1* siRNA on mRNA expression of cell cycle-related genes in RASFs, *SPACIA1*- and mock siRNA were transfected into RASFs as described in our previous report [7]. Seventy-two hours after transfection, the cells were harvested and total RNA was extracted using ISOGEN (Nippon Gene, Tokyo, Japan). DNA array analysis using the total RNA and Agilent SurePrint G3 human GE 8×60 K microarray was carried out by TaKaRa Bio Inc., Shiga, Japan. For qRT-PCR assay, cDNA was prepared from the total RNA above, using reverse transcriptase (Toyobo, Osaka, Japan) according to the manufacturer’s instructions. Target genes were amplified using an ABI 7500 Real-Time PCR System (Thermo Fisher Scientific, Waltham, MA, USA) with the following specific primers: hCDK2, 5′-cctggatgaagatggacggagc-3′ (forward) and 5′-ggcttcaagaaggctatcagagtc-3′ (reverse) (product length, 151 bp); hCDK4, 5′-tgcagtcggtggtacctgagatg-3′ (forward) and 5′-gctcaccggattaccttcatcc-3′ (reverse) (product length, 144 bp); hCDK6, 5′-gctgtctcactctagcaaccatcc-3′ (forward) and 5′-tcagagcattctgaagacagtagcc-3′ (reverse) (product length, 113 bp); hCCND1, 5′-gtacctgtaggactctcattcggg-3′ (forward) and 5′-cagcactgtgagctggcttcattg-3′ (reverse) (product length, 173 bp); hCCNE2, 5′-aggcattatgacaccaccgaaga-3′ (forward) and 5′-gaattggctagggcaatcaatcac-3′ (reverse) (product length, 165 bp); human glyceraldehyde-3-phosphate dehydrogenase (hGAPDH), 5′-gcaccgtcaaggctgagaac-3′ (forward) and 5′-atggtggtgaagacgccagt-3′ (reverse) (product length, 141 bp); hSPACIA1, 5′-ggagtactggccaagtccaagtg-3′ (forward) and 5′-ccagcagagtaggtgggtctgaa-3′ (reverse) (product length, 165 bp). Human GAPDH was utilized for normalization. Each sample was tested in triplicate, and the relative gene expression levels were normalized using the 2^−^^ΔΔCt^ method.

### 4.5. Western Blotting

To investigate CDK6 protein expression in RASFs with *SPACIA1-* or mock siRNA, Western blotting assay was performed as previously described [7], using anti-human CDK6 antibody sc-177 (Santa Cruz Biotechnology, Santa Cruz, CA, USA) and anti-human alpha-tubulin antibody T5168 (Sigma-Aldrich, St Louis, MO, USA) as the internal control.

### 4.6. Immunohistochemistry

Immunohistochemistry was performed as described in our previous report [7]. All of the synovial tissue specimens that were used in this study are from patients to undergo artificial knee joint replacement. The specimen, shown in Figure 4, is from a RA patient who treated with MTX + certolizumab pegol + prednisolone before the joint replacement. The duration of disease was 8 years. Briefly, the tissue specimens were fixed in 10% formalin and then embedded in paraffin. Deparaffinized tissue sections (5-μm thick) were antigen-retrieved by pressure cooker in 10 mM Tris-HCl (pH 9.0) and 1 mM ethylenediaminetetraacetic acid buffer for 3 min, and then incubated with anti-human CDK6 antibody sc-177 (Santa Cruz Biotechnology, Santa Cruz, CA, USA) (2 µg/mL), or normal rabbit IgG (2 µg/mL). Afterwards, the sections were rinsed and visualized by immunoperoxidase staining with the Vectastain Elite ABC kit (Vector Laboratories Ltd., Peterborough, UK) and 3,3′-diaminobenzidine tetrahydrochloride substrate. Mayer’s hematoxylin was used as a counterstain. Normal rabbit IgG and normal mouse IgG were used as negative controls.

### 4.7. Promoter Assay

To find the SPACIA1-responsible element in the proximal promoter region of the *cdk6* gene, reporter assays were performed in RASFs [12]. DNA fragments upstream of the start codon of the *cdk6* gene were amplified by PCR using the following primer sets: 5′-cgcaagcttattggagttaaacacacgcc-3′ (forward), 5′-gctccatggtcggtctagctttacttgc (reverse) (product length, 2.5 kbp). The fragment was cloned into the pGV-CS2 reporter vector (Nippon Gene) containing the luciferase gene without a promoter. Deletion constructs of 1.6 kbp and 0.9 kbp (Figure 5A,B) were obtained using internal *Kpn* I and *Bst* XI restriction enzyme sites in the 2.5 kbp fragments. Twenty-four hours after transfection of each reporter vector into RASFs, promoter activity was measured using the Luciferase Assay System (Promega, Madison, WI, USA).

### 4.8. Messenger-RNA Stability Assay

RASFs were transfected with *SPACIA1*- or mock siRNA for 72 h and treated with Actinomycin D (Sigma-Aldrich) at 1 μg/mL. The cells were harvested 0, 0.5, and 1 h after the addition of Actinomycin D. The *cdk6* mRNA levels were measured by qRT-PCR.

### 4.9. Statistical Analyses

The chi-square test (or Fisher’s exact test) was used to compare the incidence of arthritis between *SPACIA1*-deficient and wt mice (Figure 2A,C). The Mann-Whitney U test was used to compare the arthritis scores (Figure 2B,D) and the histopathological scores (Figure 2F) between *SPACIA1*-deficient and wt mice. All other statistical analyses were performed using unpaired Student’s *t*-test. The criterion for statistically significant differences was *p* < 0.05.

### 4.10. Ethical Considerations

All human (No. 1315, 7 January 2008) and animal (No. 31M0912T2, 26 December 2009) experimental protocols in this study were approved by the Ethics Review Committee of St. Marianna University School of Medicine. This study was conducted in accordance with the Declaration of Helsinki. We obtained signed consent forms from all of the patients in the study before collecting joint tissue samples from them.

## Figures and Tables

**Table 1:** Log2 ratio of G1-phase related-genes in human rheumatoid arthritis-synovial fibroblasts treated with siRNA for *SPACIA1*.

G1-phase Related-Genes	Accession Numbers	Log2 Ratio
cyclin-dependent kinase 6 (CDK6)	NM_001259	−1.6
cyclin-dependent kinase 4 (CDK4)	NM_000075	0.04
cyclin-dependent kinase 2 (CDK2)	NM_001786	−0.81
cyclin D1 (CCND1)	NM_053056	−0.07
cyclin D2 (CCND2)	NM_001759	0.29
cyclin D3 (CCND3)	NM_001760	−0.08
cyclin E1 (CCNE1)	NM_001238	0.31
cyclin E2 (CCNE2)	NM_057749	−0.86
p16 Ink4a (CDKN2A)	NM_058197	−0.11
p15 Ink4b (CDKN2B)	NM_004936	0.28
p18 Ink4c (CDKN2C)	NM_078626	−0.33
p19 Ink4d (CDKN2D)	NM_001800	0.23
p21 Cip1 (CDKN1A)	NM_078467	−0.27
p27 Kip1 (CDKN1B)	NM_004064	0.01
p57 Kip2 (CDKN1C)	NM_000076	0.17
Chk1 (CHEK1)	NM_001114121	−0.1
Chk2 (CHEK2)	NM_001005735	−0.04
cdc25A(CDC25A)	NM_001789	−0.39

## Abbreviations

CII	bovine type II collagen
CCN	cyclin
cdk (or CDK)	cyclin-dependent kinase
CIA	collagen-induced arthritis
CRE/loxP	causes recombinase/locus of crossover (x) in P1
ES	embryonic stem
FLP/frt	flippase/flippase recognition target
G1	first growth
IgG	Immunoglobulin G
hGAPDH	human glyceraldehyde-3-phosphate dehydrogenase
MCF7	Michigan Cancer Foundation 7
MTX	methotrexate
PCR	polymerase chain reaction
qRT-PCR	quantitative real time-polymerase chain reaction
RA	rheumatoid arthritis
RASFs	rheumatoid arthritis-synovial fibroblasts
SAAL1	serum amyloid A-like 1
siRNA	small interfering RNA
SPACIA1	synoviocyte proliferation-associated in collagen-induced arthritis 1
TNF	tumor necrosis factor
wt	wild-type
